# Supplementary material for: A computational model to understand mouse iron physiology and disease
Source: PLoS Comput Biol. 2019 Jan 4;15(1):e1006680. doi: 10.1371/journal.pcbi.1006680 (PMC6334977; doi:10.1371/journal.pcbi.1006680)
Supplement: S1 Text — (PDF) [file pcbi.1006680.s001.pdf]

A computational model to understand mouse iron physiology and diseases

Jignesh H. Parmar and Pedro Mendes

Center for Quantitative Medicine and Department of Cell Biology, University of Connecticut School of Medicine

Supplement

Model

The complete mathematical model is described below in detail. The model was created and simulated in the software COPASI by entering the reaction stoichiometry and kinetic rate laws for each reaction. A few derived variables are calculated with algebraic equations based on the values of the species concentrations (themselves calculated as solutions to the differential equations. Figure S1 depicts a reaction network diagram according to the SBGN standard (SBGN process diagram), Table S1 lists all parameters and their values, Table S2 the initial conditions, which correspond to the steady state when supplied an adequate diet, and finally all equations of the model are listed. The model is also included in electronic format in the supplementary ZIP file, including the native COPASI files (with the extension `.cps`) that were used to generate all of the results, SBML files (with extension `.xml`) which contain the model in a standard format that can be imported to a variety of software applications, and the data files used in parameter estimation.

Parameter Estimation

Parameter estimation was carried out on 14 unknown parameter values. Some model parameters were established by nonlinear algebraic equations as function of the 14 fitted ones; these expressions were derived from the right-hand side of the system of ODEs and assuming that the initial condition for the model is a steady state (thus the initial concentrations must be steady state concentrations). Table S1 lists the model parameters and indicates which ones were fitted versus determined from steady state equations. Table S2 lists the steady state concentrations of all species, which are also the initial concentrations (this table also lists which species initial concentrations were fitted). Figures S2-S4 plot the performance of the model fit against the experimental data for the three dietary conditions (data from Schüman *et al.* 2007).

Model equations

$\mathcal{N}$ represents the Avogadro number; "Injected" represents the total injected radioactive iron (in particle numbers). The subscript $_{PN}$ indicates that the corresponding variable represents the number of particles rather than concentration (*e.g.* Tf_{PN} for the number of particles of Tf, and [Tf] for its concentration).

$$
\begin{aligned}
\frac{\mathrm{d}\left([\mathrm{FeDuo}^{*}]\cdot Vol_{\mathrm{Duodenum}}\right)}{\mathrm{d}t} = & -\frac{\mathrm{vDuoNTBI}\cdot Vol_{\mathrm{Duodenum}}\cdot[\mathrm{FeDuo}^{*}]}{(\mathrm{KmFeFPN}+[\mathrm{FeDuo}^{*}]+[\mathrm{FeDuo}])\cdot\left(1+\frac{[\mathrm{Hepcidin}]}{\mathrm{KiHepcidinFPN}}\right)} \\
& + 2\cdot\mathrm{kInDuo}\cdot[\mathrm{Fe2Tf}^{**}]\cdot Vol_{\mathrm{Plasma}} \\
& + \mathrm{kInDuo}\cdot[\mathrm{Fe1Tf}^{*}]\cdot Vol_{\mathrm{Plasma}} \\
& + \mathrm{kInDuo}\cdot[\mathrm{Fe2Tf}^{*}]\cdot Vol_{\mathrm{Plasma}} \\
& - \mathrm{kDuoLoss}\cdot[\mathrm{FeDuo}^{*}]\cdot Vol_{\mathrm{Duodenum}}
\end{aligned}
$$

$$
\begin{aligned}
\frac{\mathrm{d}([\mathrm{FeDuo}]\cdot Vol_{\mathrm{Duodenum}})}{\mathrm{d}t} = & + \mathrm{vDiet}\cdot Vol_{\mathrm{Duodenum}} \\
& -\frac{\mathrm{vDuoNTBI}\cdot Vol_{\mathrm{Duodenum}}\cdot[\mathrm{FeDuo}]}{(\mathrm{KmFeFPN}+[\mathrm{FeDuo}]+[\mathrm{FeDuo}^{*}])\cdot\left(1+\frac{[\mathrm{Hepcidin}]}{\mathrm{KiHepcidinFPN}}\right)} \\
& - \mathrm{kDuoLoss}\cdot[\mathrm{FeDuo}]\cdot Vol_{\mathrm{Duodenum}} \\
& + 2\cdot\mathrm{kInDuo}\cdot[\mathrm{Fe2Tf}]\cdot Vol_{\mathrm{Plasma}} \\
& + \mathrm{kInDuo}\cdot[\mathrm{Fe1Tf}]\cdot Vol_{\mathrm{Plasma}} \\
& + \mathrm{kInDuo}\cdot[\mathrm{Fe2Tf}^{*}]\cdot Vol_{\mathrm{Plasma}}
\end{aligned}
$$

$$
\begin{aligned}
\frac{\mathrm{d}\left([\mathrm{FeRBC}^{*}]\cdot Vol_{\mathrm{RBC}}\right)}{\mathrm{d}t} = & - \mathrm{kRBCSpleen}\cdot[\mathrm{FeRBC}^{*}]\cdot Vol_{\mathrm{RBC}} \\
& + \mathrm{kInRBC}\cdot[\mathrm{EPO}]\cdot[\mathrm{FeBM}^{*}]\cdot Vol_{\mathrm{BoneMarrow}}
\end{aligned}
$$

$$
\begin{aligned}
\frac{\mathrm{d}([\mathrm{FeRBC}]\cdot Vol_{\mathrm{RBC}})}{\mathrm{d}t} = & - \mathrm{kRBCSpleen}\cdot[\mathrm{FeRBC}]\cdot Vol_{\mathrm{RBC}} \\
& + \mathrm{kInRBC}\cdot[\mathrm{EPO}]\cdot[\mathrm{FeBM}]\cdot Vol_{\mathrm{BoneMarrow}}
\end{aligned}
$$

$$
\begin{aligned}
\frac{\mathrm{d}\left([\mathrm{FeSpleen}^{*}]\cdot Vol_{\mathrm{Spleen}}\right)}{\mathrm{d}t} = & + \mathrm{kRBCSpleen}\cdot[\mathrm{FeRBC}^{*}]\cdot Vol_{\mathrm{RBC}} \\
& -\frac{\mathrm{vSpleenNTBI}\cdot Vol_{\mathrm{Spleen}}\cdot[\mathrm{FeSpleen}^{*}]}{(\mathrm{KmFeFPN}+[\mathrm{FeSpleen}^{*}]+[\mathrm{FeSpleen}])\cdot\left(1+\frac{[\mathrm{Hepcidin}]}{\mathrm{KiHepcidinFPN}}\right)} \\
& + \mathrm{kBMSpleen}\cdot[\mathrm{FeBM}^{*}]\cdot Vol_{\mathrm{BoneMarrow}}
\end{aligned}
$$

$$
\begin{aligned}
\frac{\mathrm{d}\left([\mathrm{FeSpleen}]\cdot Vol_{\mathrm{Spleen}}\right)}{\mathrm{d}t} = & + \mathrm{kRBCSpleen}\cdot[\mathrm{FeRBC}]\cdot Vol_{\mathrm{RBC}} \\
& -\frac{\mathrm{vSpleenNTBI}\cdot Vol_{\mathrm{Spleen}}\cdot[\mathrm{FeSpleen}]}{(\mathrm{KmFeFPN}+[\mathrm{FeSpleen}]+[\mathrm{FeSpleen}^{*}])\cdot\left(1+\frac{[\mathrm{Hepcidin}]}{\mathrm{KiHepcidinFPN}}\right)} \\
& + \mathrm{kBMSpleen}\cdot[\mathrm{FeBM}]\cdot Vol_{\mathrm{BoneMarrow}}
\end{aligned}
$$

$$
\begin{aligned}
\frac{\mathrm{d}\left([\mathrm{FeLiver}^{*}]\cdot Vol_{\mathrm{Liver}}\right)}{\mathrm{d}t} = & + \mathrm{kInLiver}\cdot[\mathrm{Fe1Tf}^{*}]\cdot Vol_{\mathrm{Plasma}} \\
& + \mathrm{kInLiver}\cdot[\mathrm{Fe2Tf}^{*}]\cdot Vol_{\mathrm{Plasma}} \\
& -\frac{\mathrm{vLiverNTBI}\cdot Vol_{\mathrm{Liver}}\cdot[\mathrm{FeLiver}^{*}]}{(\mathrm{KmFeFPN}+[\mathrm{FeLiver}^{*}]+[\mathrm{FeLiver}])\cdot\left(1+\frac{[\mathrm{Hepcidin}]}{\mathrm{KiHepcidinFPN}}\right)} \\
& + \mathrm{vNTBILiver}\cdot Vol_{\mathrm{Plasma}}\cdot\frac{[\mathrm{NTBI}^{*}]}{\mathrm{KmNTBI}+[\mathrm{NTBI}^{*}]+[\mathrm{NTBI}]}\cdot\frac{[\mathrm{NTBI}^{*}]+[\mathrm{NTBI}]}{\mathrm{KaNTBI}+[\mathrm{NTBI}^{*}]+[\mathrm{NTBI}]} \\
& + 2\cdot\mathrm{kInLiver}\cdot[\mathrm{Fe2Tf}^{**}]\cdot Vol_{\mathrm{Plasma}}
\end{aligned}
$$

$$\frac{\mathrm{d}([\mathrm{FeLiver}] \cdot Vol_{\mathrm{Liver}})}{\mathrm{d}t} = -\frac{\mathrm{vLiverNTBI} \cdot Vol_{\mathrm{Liver}} \cdot [\mathrm{FeLiver}]}{(\mathrm{KmFeFPN} + [\mathrm{FeLiver}] + [\mathrm{FeLiver}^{*}]) \cdot \left(1 + \frac{[\mathrm{Hepcidin}]}{\mathrm{KiHepcidinFPN}}\right)}$$

$$+ 2 \cdot \mathrm{kInLiver} \cdot [\mathrm{Fe2Tf}] \cdot Vol_{\mathrm{Plasma}}$$

$$+ \mathrm{kInLiver} \cdot [\mathrm{Fe1Tf}] \cdot Vol_{\mathrm{Plasma}}$$

$$+ \mathrm{kInLiver} \cdot [\mathrm{Fe2Tf}^{*}] \cdot Vol_{\mathrm{Plasma}}$$

$$+ \mathrm{vNTBILiver} \cdot Vol_{\mathrm{Plasma}} \cdot \frac{[\mathrm{NTBI}]}{\mathrm{KmNTBI} + [\mathrm{NTBI}] + [\mathrm{NTBI}^{*}]} \cdot \frac{[\mathrm{NTBI}] + [\mathrm{NTBI}^{*}]}{\mathrm{KaNTBI} + [\mathrm{NTBI}] + [\mathrm{NTBI}^{*}]}$$

$$\frac{\mathrm{d}([\mathrm{Tf}] \cdot Vol_{\mathrm{Plasma}})}{\mathrm{d}t} = + \mathrm{kInBM} \cdot [\mathrm{EPO}] \cdot [\mathrm{Fe2Tf}^{**}] \cdot Vol_{\mathrm{Plasma}}$$

$$+ \mathrm{kInDuo} \cdot [\mathrm{Fe2Tf}^{**}] \cdot Vol_{\mathrm{Plasma}}$$

$$+ \mathrm{kInLiver} \cdot [\mathrm{Fe1Tf}^{*}] \cdot Vol_{\mathrm{Plasma}}$$

$$+ \mathrm{kInBM} \cdot [\mathrm{EPO}] \cdot [\mathrm{Fe1Tf}^{*}] \cdot Vol_{\mathrm{Plasma}}$$

$$+ \mathrm{kInRest} \cdot [\mathrm{Fe1Tf}^{*}] \cdot Vol_{\mathrm{Plasma}}$$

$$+ \mathrm{kInDuo} \cdot [\mathrm{Fe1Tf}^{*}] \cdot Vol_{\mathrm{Plasma}}$$

$$+ \mathrm{kInBM} \cdot [\mathrm{EPO}] \cdot [\mathrm{Fe2Tf}] \cdot Vol_{\mathrm{Plasma}}$$

$$- \mathrm{kNTBI\_Fe1Tf} \cdot [\mathrm{NTBI}] \cdot [\mathrm{Tf}] \cdot Vol_{\mathrm{Plasma}}$$

$$+ \mathrm{kInLiver} \cdot [\mathrm{Fe2Tf}] \cdot Vol_{\mathrm{Plasma}}$$

$$+ \mathrm{kInRest} \cdot [\mathrm{Fe2Tf}] \cdot Vol_{\mathrm{Plasma}}$$

$$+ \mathrm{kInDuo} \cdot [\mathrm{Fe2Tf}] \cdot Vol_{\mathrm{Plasma}}$$

$$+ \mathrm{kInLiver} \cdot [\mathrm{Fe1Tf}] \cdot Vol_{\mathrm{Plasma}}$$

$$+ \mathrm{kInBM} \cdot [\mathrm{EPO}] \cdot [\mathrm{Fe1Tf}] \cdot Vol_{\mathrm{Plasma}}$$

$$+ \mathrm{kInRest} \cdot [\mathrm{Fe1Tf}] \cdot Vol_{\mathrm{Plasma}}$$

$$+ \mathrm{kInDuo} \cdot [\mathrm{Fe1Tf}] \cdot Vol_{\mathrm{Plasma}}$$

$$+ \mathrm{kInDuo} \cdot [\mathrm{Fe2Tf}^{*}] \cdot Vol_{\mathrm{Plasma}}$$

$$+ \mathrm{kInLiver} \cdot [\mathrm{Fe2Tf}^{*}] \cdot Vol_{\mathrm{Plasma}}$$

$$+ \mathrm{kInBM} \cdot [\mathrm{EPO}] \cdot [\mathrm{Fe2Tf}^{*}] \cdot Vol_{\mathrm{Plasma}}$$

$$+ \mathrm{kInRest} \cdot [\mathrm{Fe2Tf}^{*}] \cdot Vol_{\mathrm{Plasma}}$$

$$- \mathrm{kNTBI\_Fe1Tf} \cdot [\mathrm{NTBI}^{*}] \cdot [\mathrm{Tf}] \cdot Vol_{\mathrm{Plasma}}$$

$$- \mathrm{kdTf} \cdot [\mathrm{Tf}] \cdot Vol_{\mathrm{Plasma}}$$

$$+ \mathrm{vTf} \cdot Vol_{\mathrm{Plasma}}$$

$$+ \mathrm{kInLiver} \cdot [\mathrm{Fe2Tf}^{**}] \cdot Vol_{\mathrm{Plasma}}$$

$$+ \mathrm{kInRest} \cdot [\mathrm{Fe2Tf}^{**}] \cdot Vol_{\mathrm{Plasma}}$$

$$\begin{aligned}
\frac{\mathrm{d}([\mathrm{Fe1Tf}]\cdot Vol_{\mathrm{Plasma}})}{\mathrm{d}t} = & +\mathrm{kNTBI_Fe1Tf}\cdot[\mathrm{NTBI}]\cdot[\mathrm{Tf}]\cdot Vol_{\mathrm{Plasma}} \\
& -\mathrm{kFe1Tf_Fe2Tf}\cdot[\mathrm{Fe1Tf}]\cdot[\mathrm{NTBI}]\cdot Vol_{\mathrm{Plasma}} \\
& -\mathrm{kInLiver}\cdot[\mathrm{Fe1Tf}]\cdot Vol_{\mathrm{Plasma}} \\
& -\mathrm{kInBM}\cdot[\mathrm{EPO}]\cdot[\mathrm{Fe1Tf}]\cdot Vol_{\mathrm{Plasma}} \\
& -\mathrm{kInRest}\cdot[\mathrm{Fe1Tf}]\cdot Vol_{\mathrm{Plasma}} \\
& -\mathrm{kInDuo}\cdot[\mathrm{Fe1Tf}]\cdot Vol_{\mathrm{Plasma}} \\
& -\mathrm{kFe1Tf_Fe2Tf}\cdot[\mathrm{Fe1Tf}]\cdot[\mathrm{NTBI}^{*}]\cdot Vol_{\mathrm{Plasma}} \\
& -\mathrm{kdTf}\cdot[\mathrm{Fe1Tf}]\cdot Vol_{\mathrm{Plasma}}
\end{aligned}$$

$$\begin{aligned}
\frac{\mathrm{d}\left([\mathrm{Fe1Tf}^{*}]\cdot Vol_{\mathrm{Plasma}}\right)}{\mathrm{d}t} = & -\mathrm{kFe1Tf_Fe2Tf}\cdot[\mathrm{Fe1Tf}^{*}]\cdot[\mathrm{NTBI}^{*}]\cdot Vol_{\mathrm{Plasma}} \\
& -\mathrm{kInLiver}\cdot[\mathrm{Fe1Tf}^{*}]\cdot Vol_{\mathrm{Plasma}} \\
& -\mathrm{kInBM}\cdot[\mathrm{EPO}]\cdot[\mathrm{Fe1Tf}^{*}]\cdot Vol_{\mathrm{Plasma}} \\
& -\mathrm{kInRest}\cdot[\mathrm{Fe1Tf}^{*}]\cdot Vol_{\mathrm{Plasma}} \\
& -\mathrm{kInDuo}\cdot[\mathrm{Fe1Tf}^{*}]\cdot Vol_{\mathrm{Plasma}} \\
& -\mathrm{kFe1Tf_Fe2Tf}\cdot[\mathrm{Fe1Tf}^{*}]\cdot[\mathrm{NTBI}]\cdot Vol_{\mathrm{Plasma}} \\
& +\mathrm{kNTBI_Fe1Tf}\cdot[\mathrm{NTBI}^{*}]\cdot[\mathrm{Tf}]\cdot Vol_{\mathrm{Plasma}} \\
& -\mathrm{kdTf}\cdot[\mathrm{Fe1Tf}^{*}]\cdot Vol_{\mathrm{Plasma}}
\end{aligned}$$

$$\begin{aligned}
\frac{\mathrm{d}\left([\mathrm{Fe2Tf}^{*}]\cdot Vol_{\mathrm{Plasma}}\right)}{\mathrm{d}t} = & +\mathrm{kFe1Tf_Fe2Tf}\cdot[\mathrm{Fe1Tf}]\cdot[\mathrm{NTBI}^{*}]\cdot Vol_{\mathrm{Plasma}} \\
& +\mathrm{kFe1Tf_Fe2Tf}\cdot[\mathrm{Fe1Tf}^{*}]\cdot[\mathrm{NTBI}]\cdot Vol_{\mathrm{Plasma}} \\
& -\mathrm{kInDuo}\cdot[\mathrm{Fe2Tf}^{*}]\cdot Vol_{\mathrm{Plasma}} \\
& -\mathrm{kInLiver}\cdot[\mathrm{Fe2Tf}^{*}]\cdot Vol_{\mathrm{Plasma}} \\
& -\mathrm{kInBM}\cdot[\mathrm{EPO}]\cdot[\mathrm{Fe2Tf}^{*}]\cdot Vol_{\mathrm{Plasma}} \\
& -\mathrm{kInRest}\cdot[\mathrm{Fe2Tf}^{*}]\cdot Vol_{\mathrm{Plasma}} \\
& -\mathrm{kdTf}\cdot[\mathrm{Fe2Tf}^{*}]\cdot Vol_{\mathrm{Plasma}}
\end{aligned}$$

$$\begin{aligned}
\frac{\mathrm{d}\left([\mathrm{Fe2Tf}^{**}]\cdot Vol_{\mathrm{Plasma}}\right)}{\mathrm{d}t} = & -\mathrm{kInBM}\cdot[\mathrm{EPO}]\cdot[\mathrm{Fe2Tf}^{**}]\cdot Vol_{\mathrm{Plasma}} \\
& -\mathrm{kInDuo}\cdot[\mathrm{Fe2Tf}^{**}]\cdot Vol_{\mathrm{Plasma}} \\
& +\mathrm{kFe1Tf_Fe2Tf}\cdot[\mathrm{Fe1Tf}^{*}]\cdot[\mathrm{NTBI}^{*}]\cdot Vol_{\mathrm{Plasma}} \\
& -\mathrm{kdTf}\cdot[\mathrm{Fe2Tf}^{**}]\cdot Vol_{\mathrm{Plasma}} \\
& -\mathrm{kInLiver}\cdot[\mathrm{Fe2Tf}^{**}]\cdot Vol_{\mathrm{Plasma}} \\
& -\mathrm{kInRest}\cdot[\mathrm{Fe2Tf}^{**}]\cdot Vol_{\mathrm{Plasma}}
\end{aligned}$$

$$
\begin{aligned}
\frac{\mathrm{d}([\mathrm{Fe2Tf}]\cdot Vol_{\mathrm{Plasma}})}{\mathrm{d}t} = & -\mathrm{kInBM}\cdot[\mathrm{EPO}]\cdot[\mathrm{Fe2Tf}]\cdot Vol_{\mathrm{Plasma}} \\
& -\mathrm{kInLiver}\cdot[\mathrm{Fe2Tf}]\cdot Vol_{\mathrm{Plasma}} \\
& -\mathrm{kInRest}\cdot[\mathrm{Fe2Tf}]\cdot Vol_{\mathrm{Plasma}} \\
& -\mathrm{kInDuo}\cdot[\mathrm{Fe2Tf}]\cdot Vol_{\mathrm{Plasma}} \\
& +\mathrm{kFe1Tf\_Fe2Tf}\cdot[\mathrm{Fe1Tf}]\cdot[\mathrm{NTBI}]\cdot Vol_{\mathrm{Plasma}} \\
& -\mathrm{kdTf}\cdot[\mathrm{Fe2Tf}]\cdot Vol_{\mathrm{Plasma}}
\end{aligned}
$$

$$
\begin{aligned}
\frac{\mathrm{d}\left([\mathrm{NTBI}^*]\cdot Vol_{\mathrm{Plasma}}\right)}{\mathrm{d}t} = & +\frac{\mathrm{vDuoNTBI}\cdot Vol_{\mathrm{Duodenum}}\cdot[\mathrm{FeDuo}^*]}{(\mathrm{KmFeFPN}+[\mathrm{FeDuo}^*]+[\mathrm{FeDuo}])\cdot\left(1+\frac{[\mathrm{Hepcidin}]}{\mathrm{KiHepcidinFPN}}\right)} \\
& +\frac{\mathrm{vRestNTBI}\cdot Vol_{\mathrm{RestOfBody}}\cdot[\mathrm{FeRest}^*]}{(\mathrm{KmFeFPN}+[\mathrm{FeRest}^*]+[\mathrm{FeRest}])\cdot\left(1+\frac{[\mathrm{Hepcidin}]}{\mathrm{KiHepcidinFPN}}\right)} \\
& -\mathrm{kFe1Tf\_Fe2Tf}\cdot[\mathrm{Fe1Tf}^*]\cdot[\mathrm{NTBI}^*]\cdot Vol_{\mathrm{Plasma}} \\
& +\frac{\mathrm{vSpleenNTBI}\cdot Vol_{\mathrm{Spleen}}\cdot[\mathrm{FeSpleen}^*]}{(\mathrm{KmFeFPN}+[\mathrm{FeSpleen}^*]+[\mathrm{FeSpleen}])\cdot\left(1+\frac{[\mathrm{Hepcidin}]}{\mathrm{KiHepcidinFPN}}\right)} \\
& -\mathrm{kFe1Tf\_Fe2Tf}\cdot[\mathrm{Fe1Tf}]\cdot[\mathrm{NTBI}^*]\cdot Vol_{\mathrm{Plasma}} \\
& +\frac{\mathrm{vLiverNTBI}\cdot Vol_{\mathrm{Liver}}\cdot[\mathrm{FeLiver}^*]}{(\mathrm{KmFeFPN}+[\mathrm{FeLiver}^*]+[\mathrm{FeLiver}])\cdot\left(1+\frac{[\mathrm{Hepcidin}]}{\mathrm{KiHepcidinFPN}}\right)} \\
& -\frac{\mathrm{vNTBILiver}\cdot[\mathrm{NTBI}^*]}{\mathrm{KmNTBI}+[\mathrm{NTBI}^*]+[\mathrm{NTBI}]}\cdot\frac{[\mathrm{NTBI}^*]+[\mathrm{NTBI}]}{\mathrm{KaNTBI}+[\mathrm{NTBI}^*]+[\mathrm{NTBI}]}\cdot Vol_{\mathrm{Plasma}} \\
& -\mathrm{kNTBI\_Fe1Tf}\cdot[\mathrm{NTBI}^*]\cdot[\mathrm{Tf}]\cdot Vol_{\mathrm{Plasma}} \\
& +2\cdot\mathrm{kdTf}\cdot[\mathrm{Fe2Tf}^{**}]\cdot Vol_{\mathrm{Plasma}} \\
& +\mathrm{kdTf}\cdot[\mathrm{Fe2Tf}^*]\cdot Vol_{\mathrm{Plasma}} \\
& +\mathrm{kdTf}\cdot[\mathrm{Fe1Tf}^*]\cdot Vol_{\mathrm{Plasma}}
\end{aligned}
$$

$$
\begin{aligned}
\frac{\mathrm{d}([\mathrm{NTBI}]\cdot Vol_{\mathrm{Plasma}})}{\mathrm{d}t} = & +\frac{\mathrm{vDuoNTBI}\cdot Vol_{\mathrm{Duodenum}}\cdot[\mathrm{FeDuo}]}{(\mathrm{KmFeFPN}+[\mathrm{FeDuo}]+[\mathrm{FeDuo}^*])\cdot\left(1+\frac{[\mathrm{Hepcidin}]}{\mathrm{KiHepcidinFPN}}\right)} \\
& +\frac{\mathrm{vSpleenNTBI}\cdot Vol_{\mathrm{Spleen}}\cdot[\mathrm{FeSpleen}]}{(\mathrm{KmFeFPN}+[\mathrm{FeSpleen}]+[\mathrm{FeSpleen}^*])\cdot\left(1+\frac{[\mathrm{Hepcidin}]}{\mathrm{KiHepcidinFPN}}\right)} \\
& +\frac{\mathrm{vLiverNTBI}\cdot Vol_{\mathrm{Liver}}\cdot[\mathrm{FeLiver}]}{(\mathrm{KmFeFPN}+[\mathrm{FeLiver}]+[\mathrm{FeLiver}^*])\cdot\left(1+\frac{[\mathrm{Hepcidin}]}{\mathrm{KiHepcidinFPN}}\right)} \\
& -\mathrm{kNTBI\_Fe1Tf}\cdot[\mathrm{NTBI}]\cdot[\mathrm{Tf}]\cdot Vol_{\mathrm{Plasma}} \\
& +\frac{\mathrm{vRestNTBI}\cdot Vol_{\mathrm{RestOfBody}}\cdot[\mathrm{FeRest}]}{(\mathrm{KmFeFPN}+[\mathrm{FeRest}]+[\mathrm{FeRest}^*])\cdot\left(1+\frac{[\mathrm{Hepcidin}]}{\mathrm{KiHepcidinFPN}}\right)} \\
& -\mathrm{kFe1Tf\_Fe2Tf}\cdot[\mathrm{Fe1Tf}]\cdot[\mathrm{NTBI}]\cdot Vol_{\mathrm{Plasma}} \\
& -\mathrm{kFe1Tf\_Fe2Tf}\cdot[\mathrm{Fe1Tf}^*]\cdot[\mathrm{NTBI}]\cdot Vol_{\mathrm{Plasma}} \\
& -\frac{\mathrm{vNTBILiver}\cdot[\mathrm{NTBI}]}{\mathrm{KmNTBI}+[\mathrm{NTBI}]+[\mathrm{NTBI}^*]}\cdot\frac{[\mathrm{NTBI}]+[\mathrm{NTBI}^*]}{\mathrm{KaNTBI}+[\mathrm{NTBI}]+[\mathrm{NTBI}^*]}\cdot Vol_{\mathrm{Plasma}} \\
& +2\cdot\mathrm{kdTf}\cdot[\mathrm{Fe2Tf}]\cdot Vol_{\mathrm{Plasma}} \\
& +\mathrm{kdTf}\cdot[\mathrm{Fe2Tf}^*]\cdot Vol_{\mathrm{Plasma}} \\
& +\mathrm{kdTf}\cdot[\mathrm{Fe1Tf}]\cdot Vol_{\mathrm{Plasma}}
\end{aligned}
$$

$$
\begin{aligned}
\frac{\mathrm{d}\left([\mathrm{FeBM}^{*}]\cdot Vol_{\mathrm{BoneMarrow}}\right)}{\mathrm{d}t} &= +2\cdot \mathrm{kInBM}\cdot[\mathrm{EPO}]\cdot[\mathrm{Fe2Tf}^{**}]\cdot Vol_{\mathrm{Plasma}} \\
&\quad +\mathrm{kInBM}\cdot[\mathrm{EPO}]\cdot[\mathrm{Fe1Tf}^{*}]\cdot Vol_{\mathrm{Plasma}} \\
&\quad +\mathrm{kInBM}\cdot[\mathrm{EPO}]\cdot[\mathrm{Fe2Tf}^{*}]\cdot Vol_{\mathrm{Plasma}} \\
&\quad -\mathrm{kInRBC}\cdot[\mathrm{EPO}]\cdot[\mathrm{FeBM}^{*}]\cdot Vol_{\mathrm{BoneMarrow}} \\
&\quad -\mathrm{kBMSpleen}\cdot[\mathrm{FeBM}^{*}]\cdot Vol_{\mathrm{BoneMarrow}}
\end{aligned}
$$

$$
\begin{aligned}
\frac{\mathrm{d}([\mathrm{FeBM}]\cdot Vol_{\mathrm{BoneMarrow}})}{\mathrm{d}t} &= +2\cdot \mathrm{kInBM}\cdot[\mathrm{EPO}]\cdot[\mathrm{Fe2Tf}]\cdot Vol_{\mathrm{Plasma}} \\
&\quad +\mathrm{kInBM}\cdot[\mathrm{EPO}]\cdot[\mathrm{Fe1Tf}]\cdot Vol_{\mathrm{Plasma}} \\
&\quad +\mathrm{kInBM}\cdot[\mathrm{EPO}]\cdot[\mathrm{Fe2Tf}^{*}]\cdot Vol_{\mathrm{Plasma}} \\
&\quad -\mathrm{kInRBC}\cdot[\mathrm{EPO}]\cdot[\mathrm{FeBM}]\cdot Vol_{\mathrm{BoneMarrow}} \\
&\quad -\mathrm{kBMSpleen}\cdot[\mathrm{FeBM}]\cdot Vol_{\mathrm{BoneMarrow}}
\end{aligned}
$$

$$
\begin{aligned}
\frac{\mathrm{d}\left([\mathrm{FeRest}^{*}]\cdot Vol_{\mathrm{RestOfBody}}\right)}{\mathrm{d}t} &= -\frac{\mathrm{vRestNTBI}\cdot Vol_{\mathrm{RestOfBody}}\cdot[\mathrm{FeRest}^{*}]}{(\mathrm{KmFeFPN}+[\mathrm{FeRest}^{*}]+[\mathrm{FeRest}])\cdot\left(1+\frac{[\mathrm{Hepcidin}]}{\mathrm{KiHepcidinFPN}}\right)} \\
&\quad +\mathrm{kInRest}\cdot[\mathrm{Fe1Tf}^{*}]\cdot Vol_{\mathrm{Plasma}} \\
&\quad +\mathrm{kInRest}\cdot[\mathrm{Fe2Tf}^{*}]\cdot Vol_{\mathrm{Plasma}} \\
&\quad +2\cdot\mathrm{kInRest}\cdot[\mathrm{Fe2Tf}^{**}]\cdot Vol_{\mathrm{Plasma}} \\
&\quad -\mathrm{kRestLoss}\cdot[\mathrm{FeRest}^{*}]\cdot Vol_{\mathrm{RestOfBody}}
\end{aligned}
$$

$$
\begin{aligned}
\frac{\mathrm{d}\left([\mathrm{FeRest}]\cdot Vol_{\mathrm{RestOfBody}}\right)}{\mathrm{d}t} &= +2\cdot\mathrm{kInRest}\cdot[\mathrm{Fe2Tf}]\cdot Vol_{\mathrm{Plasma}} \\
&\quad -\frac{\mathrm{vRestNTBI}\cdot Vol_{\mathrm{RestOfBody}}\cdot[\mathrm{FeRest}]}{(\mathrm{KmFeFPN}+[\mathrm{FeRest}]+[\mathrm{FeRest}^{*}])\cdot\left(1+\frac{[\mathrm{Hepcidin}]}{\mathrm{KiHepcidinFPN}}\right)} \\
&\quad +\mathrm{kInRest}\cdot[\mathrm{Fe1Tf}]\cdot Vol_{\mathrm{Plasma}} \\
&\quad +\mathrm{kInRest}\cdot[\mathrm{Fe2Tf}^{*}]\cdot Vol_{\mathrm{Plasma}} \\
&\quad -\mathrm{kRestLoss}\cdot[\mathrm{FeRest}]\cdot Vol_{\mathrm{RestOfBody}}
\end{aligned}
$$

$$
\begin{aligned}
\frac{\mathrm{d}\left([\mathrm{FeOutside}^{*}]\cdot Vol_{\mathrm{RestOfBody}}\right)}{\mathrm{d}t} &= +\mathrm{kDuoLoss}\cdot[\mathrm{FeDuo}^{*}]\cdot Vol_{\mathrm{Duodenum}} \\
&\quad +\mathrm{kRestLoss}\cdot[\mathrm{FeRest}^{*}]\cdot Vol_{\mathrm{RestOfBody}}
\end{aligned}
$$

$$
\begin{aligned}
\frac{\mathrm{d}([\mathrm{Hepcidin}]\cdot Vol_{\mathrm{Plasma}})}{\mathrm{d}t} &= +\frac{\mathrm{ksHepcidin}\cdot\mathrm{FeTftotal}\cdot\mathrm{KEPOHepcidin}^{\mathrm{hEPOHepcidin}}}{\mathrm{KEPOHepcidin}^{\mathrm{hEPOHepcidin}}+[\mathrm{EPO}]^{\mathrm{hEPOHepcidin}}}\cdot Vol_{\mathrm{Plasma}} \\
&\quad -\mathrm{kdHepcidin}\cdot[\mathrm{Hepcidin}]\cdot Vol_{\mathrm{Plasma}}
\end{aligned}
$$

$$
\begin{aligned}
\frac{\mathrm{d}([\mathrm{EPO}]\cdot Vol_{\mathrm{Plasma}})}{\mathrm{d}t} &= +\frac{\mathrm{vEPO}\cdot\mathrm{KiEPORBC}^{\mathrm{hEPO}}}{\mathrm{KiEPORBC}^{\mathrm{hEPO}}+([\mathrm{FeRBC}]+[\mathrm{FeRBC}^{*}])^{\mathrm{hEPO}}}\cdot Vol_{\mathrm{Plasma}} \\
&\quad -\mathrm{kdEPO}\cdot[\mathrm{EPO}]\cdot Vol_{\mathrm{Plasma}}
\end{aligned}
$$

$$\text{Total\_Fe}^*_{\text{PN}} = \text{FeDuo}^*_{\text{PN}} + \text{FeLiver}^*_{\text{PN}} + \text{FeSpleen}^*_{\text{PN}} + \text{FeRBC}^*_{\text{PN}} + \text{FeRest}^*_{\text{PN}} + \text{Fe2Tf}^*_{\text{PN}} + \text{NTBI}^*_{\text{PN}} + 2 \cdot \text{Fe2Tf}^{**}_{\text{PN}} + \text{Fe1Tf}^*_{\text{PN}} + \text{FeBM}^*_{\text{PN}}$$

$$\text{Total\_Fe}_{\text{PN}} = \text{FeDuo}_{\text{PN}} + \text{FeLiver}_{\text{PN}} + \text{FeSpleen}_{\text{PN}} + \text{FeRBC}_{\text{PN}} + \text{FeRest}_{\text{PN}} + 2 \cdot \text{Fe2Tf}_{\text{PN}} + \text{Fe2Tf}^*_{\text{PN}} + \text{Fe1Tf}_{\text{PN}} + \text{NTBI}_{\text{PN}} + \text{FeBM}_{\text{PN}}$$

$$\text{Total\_Fe (g)} = \frac{\text{Total\_Fe} \cdot 55.845}{\mathcal{N}}$$

$$[\text{Total\_Fe}] = \frac{\text{FeDuo}_{\text{PN}} + \text{FeLiver}_{\text{PN}} + \text{FeSpleen}_{\text{PN}} + \text{FeRBC}_{\text{PN}} + \text{FeRest}_{\text{PN}} + 2 \cdot \text{Fe2Tf}_{\text{PN}} + \text{Fe2Tf}^*_{\text{PN}} + \text{Fe1Tf}_{\text{PN}} + \text{NTBI}_{\text{PN}} + \text{FeBM}_{\text{PN}}}{\mathcal{N} \cdot \left(Vol_{\text{Duodenum}} + Vol_{\text{Liver}} + Vol_{\text{Plasma}} + Vol_{\text{RBC}} + Vol_{\text{RestOfBody}} + Vol_{\text{Spleen}}\right)}$$

$$\text{FePlasma}^*_{\text{PN}} = \text{Fe2Tf}^*_{\text{PN}} + \text{NTBI}^*_{\text{PN}} + \text{Fe1Tf}^*_{\text{PN}} + 2 \cdot \text{Fe2Tf}^{**}_{\text{PN}}$$

$$\text{FePlasma}_{\text{PN}} = 2 \cdot \text{Fe2Tf}_{\text{PN}} + \text{Fe2Tf}^*_{\text{PN}} + \text{Fe1Tf}_{\text{PN}} + \text{NTBI}_{\text{PN}}$$

$$[\text{FePlasma}^*] = \frac{\text{FePlasma}^*}{Vol_{\text{Plasma}} \cdot \mathcal{N}}$$

$$[\text{FePlasma}] = \frac{\text{FePlasma}}{Vol_{\text{Plasma}} \cdot \mathcal{N}}$$

$$\text{Total\_Tf}_{\text{PN}} = \text{Tf}_{\text{PN}} + \text{Fe1Tf}^*_{\text{PN}} + \text{Fe1Tf}_{\text{PN}} + \text{Fe2Tf}^{**}_{\text{PN}} + \text{Fe2Tf}^*_{\text{PN}} + \text{Fe2Tf}_{\text{PN}}$$

$$[\text{Total\_Tf}] = \frac{\text{Total\_Tf}}{\mathcal{N} \cdot Vol_{\text{Plasma}}}$$

$$[\text{Total\_Tf}]\ (\text{mg/ml}) = [\text{Total\_Tf}] \cdot 76000$$

$$\text{TfSaturation (\%)} = \frac{100 \cdot \left(2 \cdot \text{Fe2Tf}^*_{\text{PN}} + 2 \cdot \text{Fe2Tf}_{\text{PN}} + \text{Fe1Tf}^*_{\text{PN}} + \text{Fe1Tf}_{\text{PN}} + 2 \cdot \text{Fe2Tf}^{**}_{\text{PN}}\right)}{2 \cdot \left(\text{Fe2Tf}^*_{\text{PN}} + \text{Fe1Tf}^*_{\text{PN}} + \text{Tf}_{\text{PN}} + \text{Fe2Tf}^{**}_{\text{PN}} + \text{Fe2Tf}_{\text{PN}} + \text{Fe1Tf}_{\text{PN}}\right)}$$

$$[\text{Total\_FeTf}] = 2 \cdot [\text{Fe2Tf}] + [\text{Fe1Tf}] + [\text{Fe1Tf}^*] + 2 \cdot [\text{Fe2Tf}^{**}] + 2 \cdot [\text{Fe2Tf}^*]$$

$$\text{PDuo} = \frac{100 \cdot \text{FeDuo}^*_{\text{PN}}}{\text{Injected}}$$

$$\text{PLiver} = \frac{100 \cdot \text{FeLiver}^*_{\text{PN}}}{\text{Injected}}$$

$$\text{PSpleen} = \frac{100 \cdot \text{FeSpleen}^*_{\text{PN}}}{\text{Injected}}$$

$$\text{PRBC} = \frac{100 \cdot \text{FeRBC}^*_{\text{PN}}}{\text{Injected}}$$

$$\text{PBM} = \frac{100 \cdot \text{FeBM}^*_{\text{PN}}}{\text{Injected}}$$

$$\text{PPlasma} = \frac{100 \cdot \left(\text{NTBI}^*_{\text{PN}} + 2 \cdot \text{Fe2Tf}^{**}_{\text{PN}} + \text{Fe2Tf}^*_{\text{PN}} + \text{Fe1Tf}^*_{\text{PN}}\right)}{\text{Injected}}$$

$$\text{PRest} = \frac{100 \cdot \text{FeRest}^*_{\text{PN}}}{\text{Injected}}$$

$$\text{POutside} = \frac{100 \cdot \text{FeOutside}^*_{\text{PN}}}{\text{Injected}}$$

$$\text{Injected} = 3.073 \cdot 10^{15}$$
