# Supplementary material for: A computational model to understand mouse iron physiology and disease
Source: PLoS Comput Biol. 2019 Jan 4;15(1):e1006680. doi: 10.1371/journal.pcbi.1006680 (PMC6334977; doi:10.1371/journal.pcbi.1006680)
Supplement: S1 Table — (PDF) [file pcbi.1006680.s002.pdf]

S1 Table – List of all model parameters including their values, units, and a brief description of their role.

Parameter	Status[1]	Value	Unit	Description
$Vol_{Duodenum}$	○	3.84615×10^{-5}	*l*	Volume of the duodenum compartment
Vol_{RBC}	○	7.9×10^{-4}	*l*	Volume of the RBC compartment
Vol_{Spleen}	○	6.73077×10^{-5}	*l*	Volume of the spleen compartment
Vol_{Liver}	○	1.161905×10^{-3}	*l*	Volume of the liver compartment
Vol_{Plasma}	○	1.3×10^{-3}	*l*	Volume of the plasma compartment
$Vol_{BoneMarrow}$	○	2.14286×10^{-4}	*l*	Volume of the bone marrow compartment
$Vol_{RestOfBody}$	○	1.96948×10^{-2}	*l*	Volume of the "rest of body" compartment
kInDuo	▲	0.397138	d^{-1}	1st order rate constant of iron transfer from Transferrin to Duodenum
kInLiver	▲	2.11666	d^{-1}	1st order rate constant of iron transfer from Transferrin to Liver
kInRBC	†	5.03844×10^{11}	d^{-1}	1st order rate constant of iron transfer from Bone Marrow to RBC
kInRest	†	4.78121	d^{-1}	1st order rate constant of iron transfer from Transferrin to "Rest of Body"
kInBM	†	4.06878×10^{12}	d^{-1}	1st order rate constant of iron transfer from Transferrin to Bone Marrow
kDuoLoss	▲	6.80738×10^{-5}	d^{-1}	1st order rate constant of iron loss from Duodenum compartment
kRestLoss	†	0.0168620	d^{-1}	1st order rate constant of iron loss from "Rest of Body" compartment
kBMSpleen	†	0.103218	d^{-1}	1st order rate constant for transfer or iron from Bone Marrow to Spleen
VDuoNTBI	†	0.200906	***mol*** d^{-1}	Maximal rate of Ferroportin in Duodenum compartment (FPN *Vmax*)
VLiverNTBI	†	0.0444795	***mol*** d^{-1}	Maximal rate of Ferroportin in Liver compartment (FPN *Vmax*)
VSpleenNTBI	†	2.06738	***mol*** d^{-1}	Maximal rate of Ferroportin in Spleen compartment (FPN *Vmax*)
VRestNTBI	▲	0.0101453	***mol*** d^{-1}	Maximal rate of Ferroportin in Rest of Body compartment (FPN *Vmax*)
kRBCSpleen	○	0.03	d^{-1}	Steady state rate of transfer of iron from RBC to Spleen
KmFeFPN	▲	0.112511	***M***	Michaelis constant of Ferroportin reactions
KiHepcidinFPN	○	6.3×10^{-9}	***M***	Inhibition constant of Ferroportin reactions by Hepcidin
vDiet	†	3.46965×10^{-3}	***M*** d^{-1}	Steady state rate of iron entry into duodenum (from diet)
fDiet	▲<br>○<br>▲	1.29060×10^{-6}<br>1<br>3.11813		Factor to adjust *vDiet* to the different levels of iron supply (deficient, adequate, and rich iron, in this order). The effective rate of dietary iron entry is the product *fDiet*×*vDiet*
kNTBI_Fe1Tf	†	1.00400×10^{9}	$M^{-1}d^{-1}$	Second order rate constant of NTBI binding to apoTransferrin
kFe1Tf_Fe2Tf	○	1.00400×10^{9}	$M^{-1}d^{-1}$	Second order rate constant of NTBI binding to Fe1Tf
ksHepcidin	†	3.98766×10^{-4}	d^{-1}	1st order activation constant of Hepcidin synthesis by TotalFeTf
KEPOHepcidin	○	5.0×10^{-12}	***M***	Inhibition constant of EPO on Hepcidin synthesis
hEPOHepcidin	○	4		Hill coefficient for inhibition of EPO on Hepcidin synthesis
kdHepcidin	○	0.75616	d^{-1}	v order rate constant for degradation of Hepcidin
VNTBILiver	▲	14.1511	***M*** d^{-1}	Maximal rate of import of NTBI into the liver (ZIP14 *Vmax*)
KmNTBI	▲	6.79291×10^{-4}	***M***	Michaelis constant for NTBI entry into the liver (ZIP14 *Km*)

KaNTBI	▲	2.55016×10^{-4}	***M***	Substrate activation constant for NTBI entry into the liver
vEPO	†	2.62675×10^{-9}	***M d⁻¹***	Maximal rate of synthesis of EPO (when [FeRBC]=0)
hEPO	○	6.5		Hill coefficient for the inhibition of EPO synthesis by FeRBC
KiEPORBC	○	0.01	***M***	Inhibition constant of EPO synthesis by FeRBC
kdEPO	○	4.8	d^{-1}	1st order rate constant for the degradation of EPO
vTf	†	1.54710×10^{-5}	$M\ d^{-1}$	Rate of synthesis of Transferrin
kdTf	○	0.4	d^{-1}	1st order rate constant of degradation of Transferrin
Injected	○	3.073×10^{15}		Number of radioactive ferrous ions injected (tracer)

[1]- parameters marked with a triangle (▲) were fit to the data, those marked with a dagger (†) were determined by steady state equations, and those marked with an open circle (○) were determined by other data or assumptions.
