# Supplementary material for: A computational model to understand mouse iron physiology and disease
Source: PLoS Comput Biol. 2019 Jan 4;15(1):e1006680. doi: 10.1371/journal.pcbi.1006680 (PMC6334977; doi:10.1371/journal.pcbi.1006680)
Supplement: S2 Table — This is also the initial condition for most of the simulations. (PDF) [file pcbi.1006680.s003.pdf]

S2 Table – List of model variables and their values in the steady state achieved with an adequate iron diet (*vDiet*=3.4484×10^{-3} M d^{-1} and *fDiet*=1). This is also the initial condition for most of the simulations.

Variable	Status[1]	Value	Unit	Description
**[*FeRBC*]**	†	23	***mM***	Concentration of iron in RBC (iron mass = 0.997 *mg*)
**[*FeDuo*]**	▲	10.0222	***mM***	Concentration of iron in duodenum (iron mass = 0.0218 *mg*)
**[*FeSpleen*]**	▲	2.26838	***mM***	Concentration of iron in spleen (iron mass = 0.00823 *mg*)
**[*FeBM*]**	▲	2.08051	***mM***	Concentration of iron in bone marrow (iron mass = 0.0257 *mg*)
**[*FeLiver*]**	†	1.3	***mM***	Concentration of iron in liver (iron mass = 0.0843 *mg*)
**[*FeRest*]**	†	0.40176	***mM***	Concentration of iron in “rest of body” (iron mass = 0.444 *mg*)
**[*Fe2Tf*]**	†	0.0189625	***mM***	Concentration of Fe2Tf species (2 Fe^{3+} bound to Tf) (iron mass = 0.00270 *mg*)
**[*Tf*]**	†	0.0116103	***mM***	Concentration of free transferrin
**[*Fe1Tf*]**	†	8.12714	***µM***	Concentration of Fe1Tf species (1 Fe^{3+} bound to Tf) (iron mass = 0.0006 *mg*)
**[*NTBI*]**	†	40	***nM***	Concentration of NTBI (iron mass = 2.893 *ng*)
**[*Hepcidin*]**	†	23.00092	***nM***	Concentration of Hepcidin
**[*EPO*]**	†	2.4267	***pM***	Concentration of Erythropoietin
**[*All tracer species*]**		0	***mM***	Concentration of all tracer species (FeDuo*, FeLiver*, FeRBC*, FeS-pleen*, FeRest*, Fe1Tf*, Fe2Tf*, Fe2Tf**, NTBI*)
**[*FePlasma*]**	○	0.0460921	***mM***	Total concentration of iron in plasma, sum of *NTBI* with all Tf-bound iron (iron mass = 0.00329 *mg*)
**[*TotalTf*]**	○	0.0386999	***mM***	Total concentration of transferrin with and without iron bound (2.945 *mg/ml*)
**[*TotalFe*]**	○	1.59923	***mM***	Total iron concentration in the body (iron mass = 1.6 *mg*)
$\boldsymbol{Tf_{sat}}$	○	59.499	***%***	Transferrin saturation (percentage of iron binding sites occupied in Tf)
FeRBC transition time	○	33.3	***d***	Time for iron to transition through the RBC compartment (essentially the same as the average RBC lifetime)

[1] – items marked with a triangle (▲) were fit to the data, those marked with a dagger (†) were determined by steady state equations, and those marked with an open circle (○) were determined by other data or assumptions.
