# Supplementary figures and images for: A computational model to understand mouse iron physiology and disease
Source: PLoS Comput Biol. 2019 Jan 4;15(1):e1006680. doi: 10.1371/journal.pcbi.1006680 (PMC6334977; doi:10.1371/journal.pcbi.1006680)

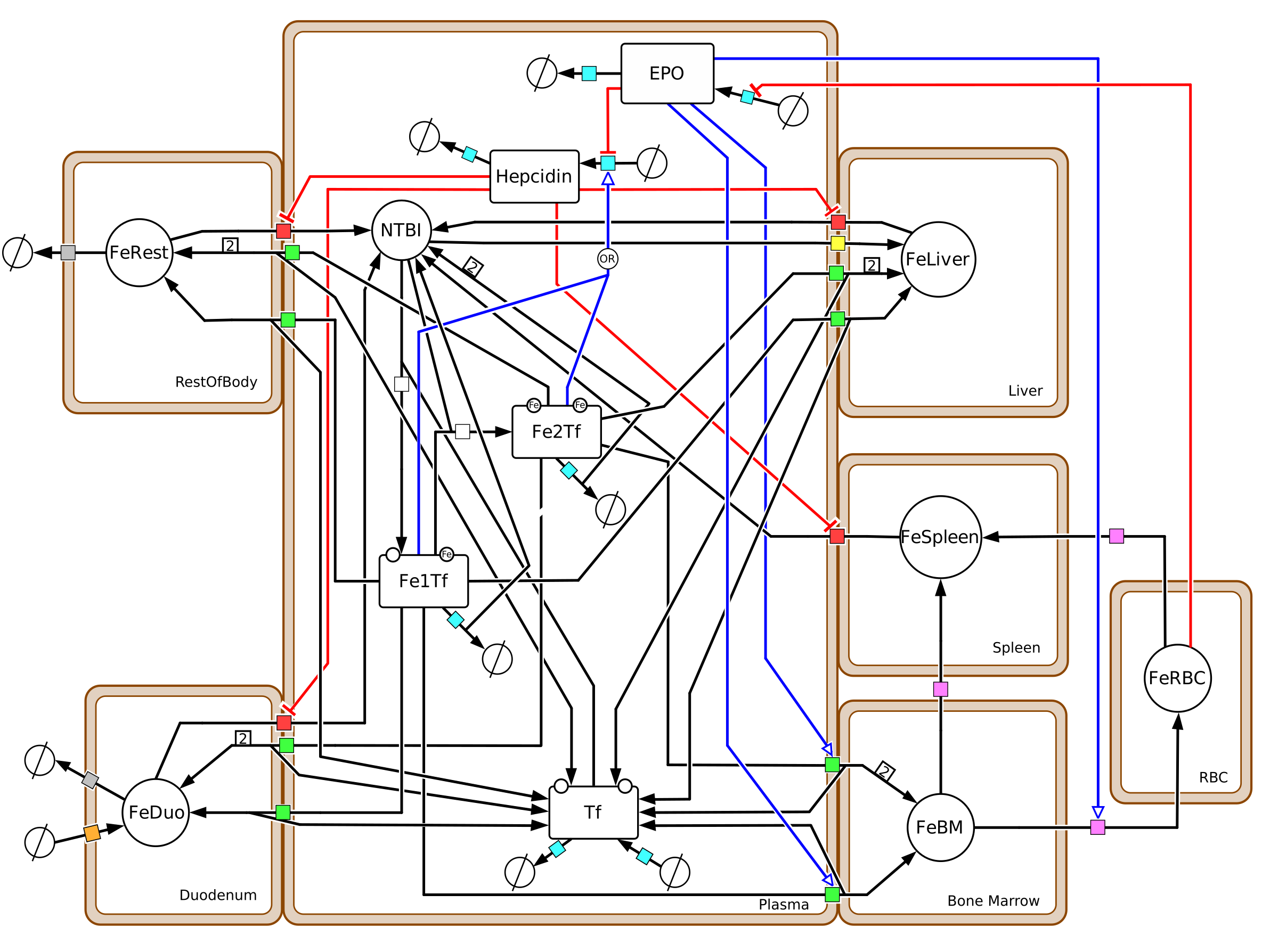

Supplement: S1 Fig — The diagram follows the SBGN standard. Black arrows represent reactions, blue arrows activation, and red arrows inhibition. Reactions boxes in red represent iron export to plasma through ferroportin, in green iron transfer from transferrin into cells (through the transferrin receptors), in yellow the import of NTBI from plasma to the liver, in cyan protein synthesis and degradation, in magenta the processes of erythroid cell dynamics, in grey iron loss from the body, and in orange dietary iron entry to the duodenum. The symbol “OR” indicates that the activation is by the sum of the concentrations of the two iron-loaded transferrin forms. (TIF) [file pcbi.1006680.s005.tif]

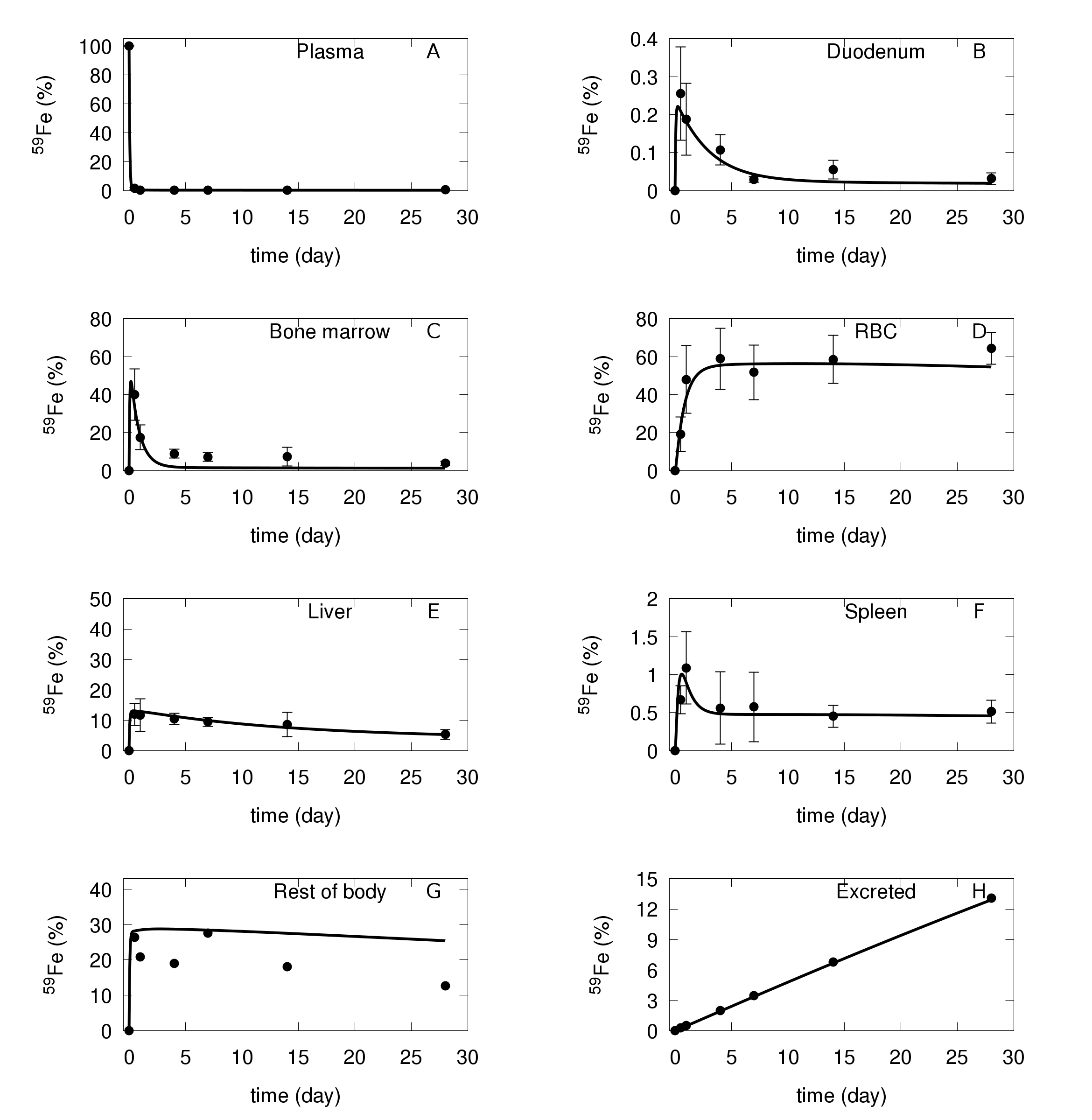

Supplement: S2 Fig — Continuous lines represent the model fit, while filled circles represent the data from Schüman et al. (2007). Vertical bars represent one standard deviation. The abscissa represents the proportion of total injected 59Fe, the ordinate is time after injection. A) plasma, B) duodenum, C) bone marrow, D) red blood cells, E) liver, F) spleen, G) rest of body, and H) excreted. Note that the standard deviation in panel A is smaller than the size of the symbols, while in panels G and H it was not displayed as it is very large (due to these data being algebraic sums of various terms). (TIF) [file pcbi.1006680.s006.tif]

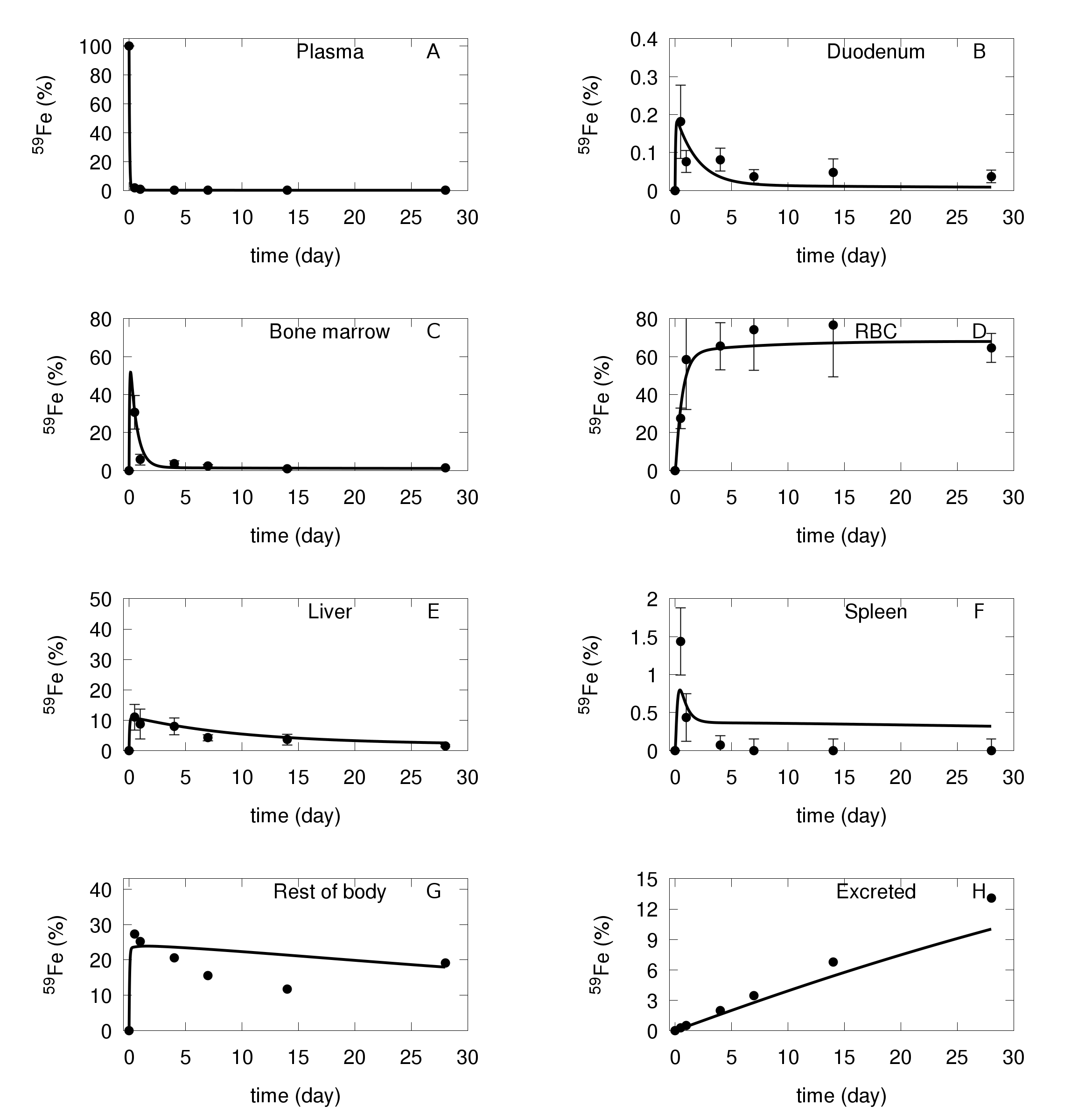

Supplement: S3 Fig — Continuous lines represent the model fit, while filled circles represent the data from Schüman et al. (2007). Vertical bars represent one standard deviation. The abscissa represents the proportion of total injected 59Fe, the ordinate is time after injection. A) plasma, B) duodenum, C) bone marrow, D) red blood cells, E) liver, F) spleen, G) rest of body, and H) excreted. Note that the standard deviation in panel A is smaller than the size of the symbols, while in panels G and H it was not displayed as it is very large (due to these data being algebraic sums of various terms). (TIF) [file pcbi.1006680.s007.tif]

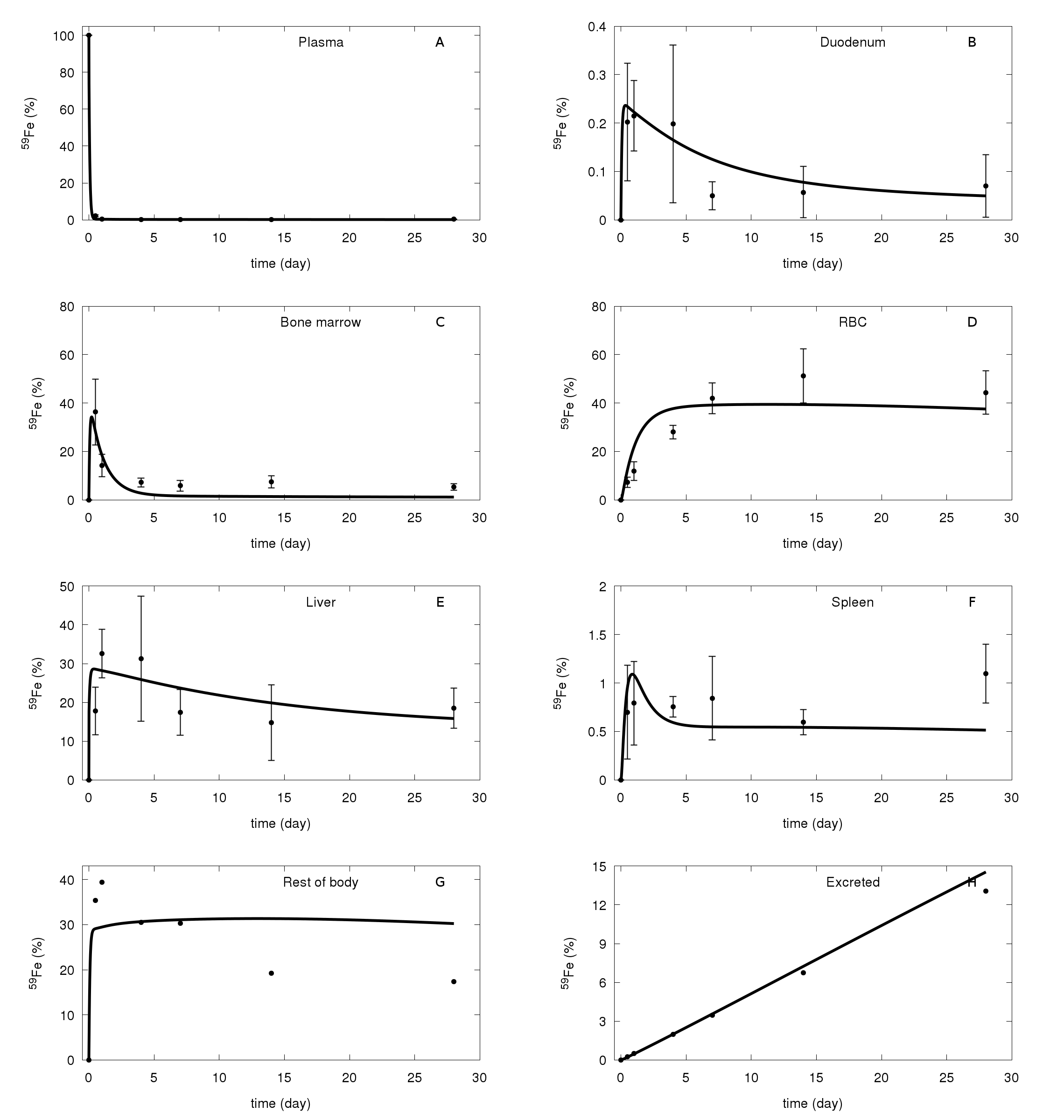

Supplement: S4 Fig — Continuous lines represent the model fit, while filled circles represent the data from Schüman et al. (2007). Vertical bars represent one standard deviation. The abscissa represents the proportion of total injected 59Fe, the ordinate is time after injection. A) plasma, B) duodenum, C) bone marrow, D) red blood cells, E) liver, F) spleen, G) rest of body, and H) excreted. Note that the standard deviation in panel A is smaller than the size of the symbols, while in panels G and H it was not displayed as it is very large (due to these data being algebraic sums of various terms). (TIF) [file pcbi.1006680.s008.tif]

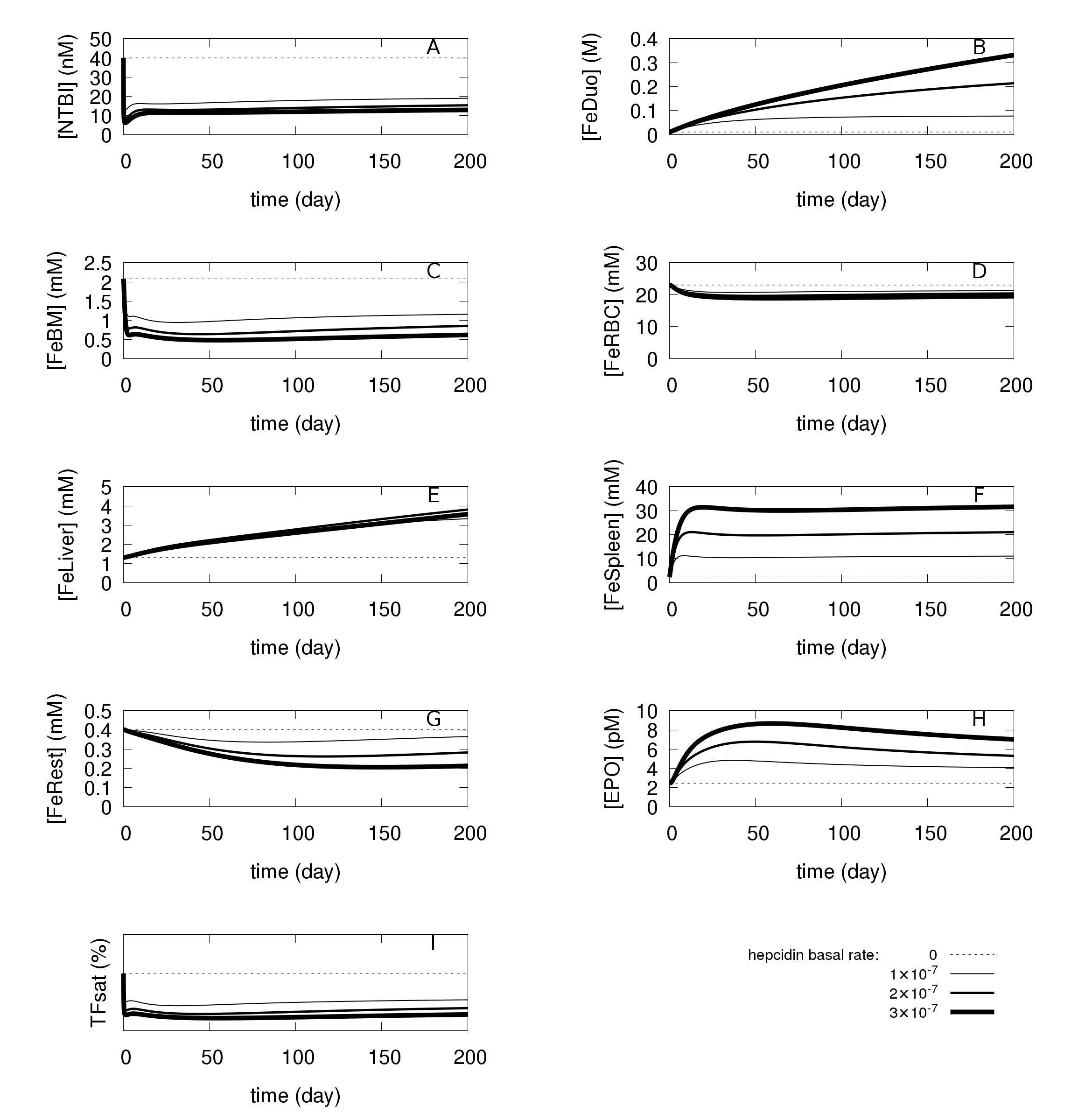

Supplement: S5 Fig — At time zero the basal rate of hepcidin synthesis is raised (from 0 to 10−7, 2×10^−7^, or 3×10^−7^ M/day) and the time course is followed for 200 days. Panels A-H display the relative change versus the case without elevated hepcidin basal rate (WT control). A: NTBI, B: iron in duodenum, C: iron in bone marrow, D: iron in RBC, E: iron in liver, F: iron in spleen, G: iron in rest of body, H: erythropoietin, I: transferrin saturation (%). (TIF) [file pcbi.1006680.s009.tif]

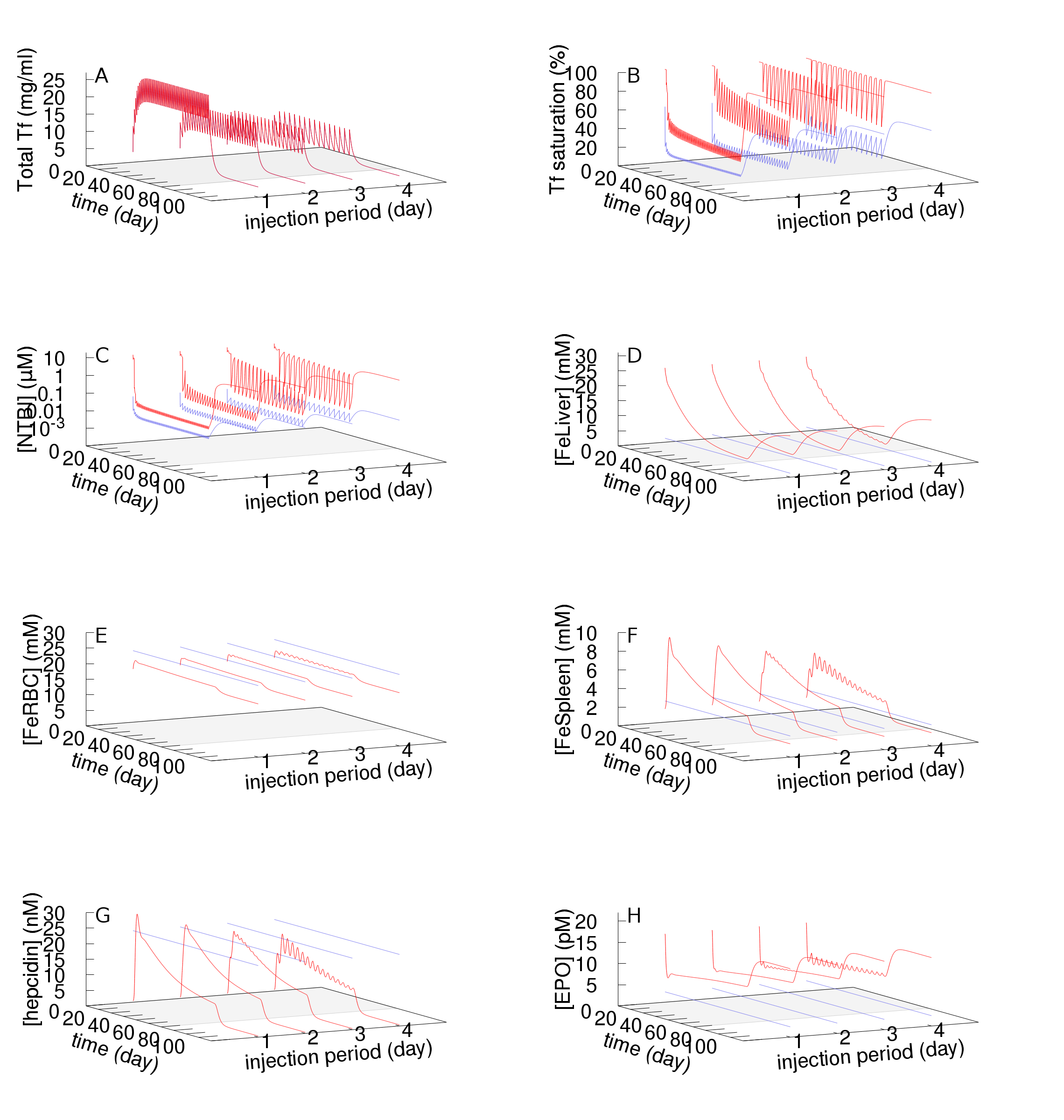

Supplement: S6 Fig — Injections of apo-transferrin were simulated by increasing apo-transferrin plasma concentration by 102 μM every day, every 2, every 3 or every 4 days for a duration of 60 days (shaded area). This procedure is applied both to the wild type model (blue lines) and to the thalassemia major model (red lines). The thalassemia major model is initialized by reducing parameter kInBM to 25% WT value, and kRBCSpleen increased to 400% WT value, then a time course of 365 days is run prior to the application of the transferrin treatment (not shown on this figure). A–total transferrin; B–transferrin saturation; C–NTBI concentration; D–liver iron concentration; E–RBC iron concentration; F–Spleen iron concentration; G–hepcidin concentration; H–EPO concentration. (TIF) [file pcbi.1006680.s010.tif]

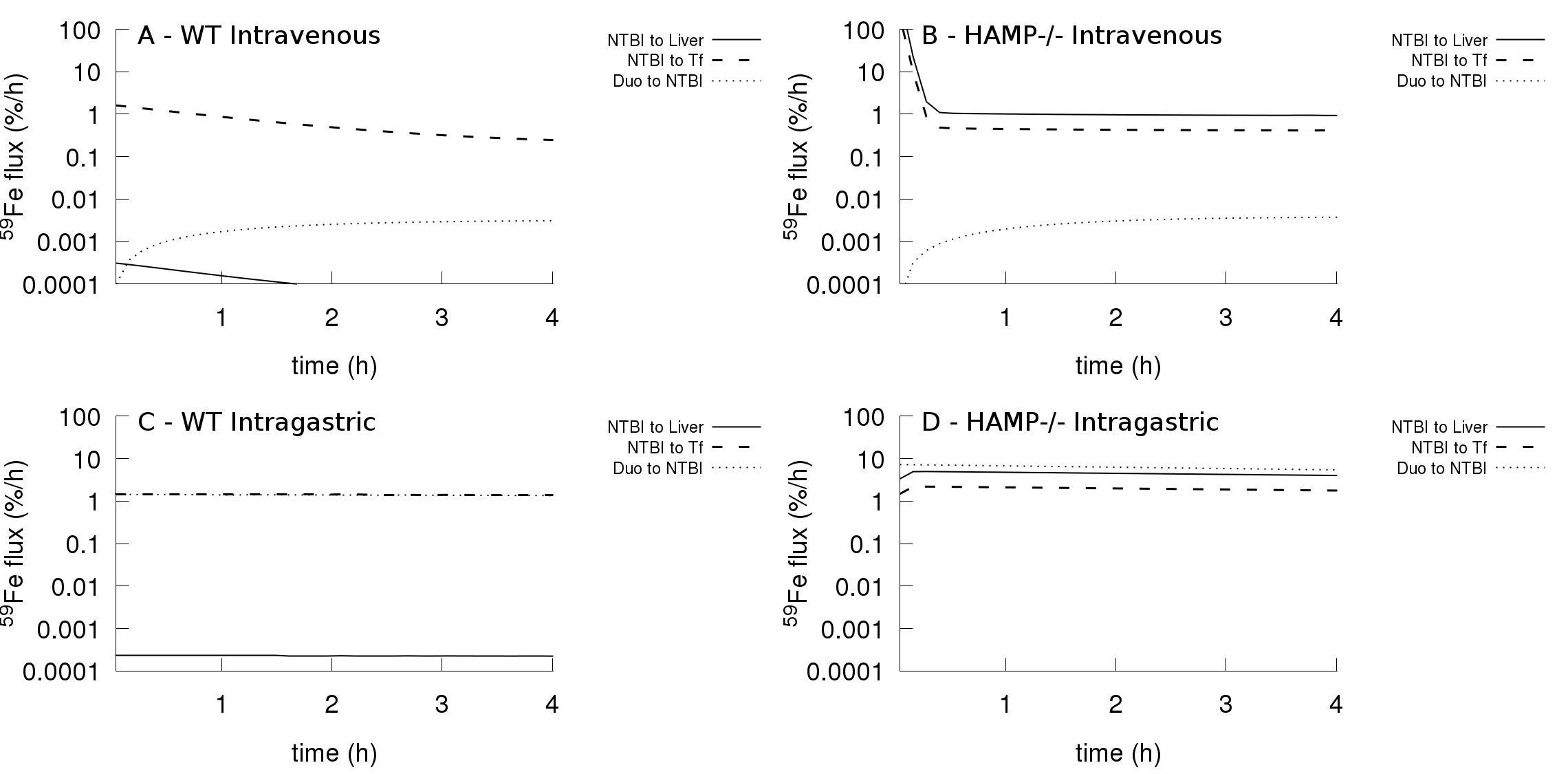

Supplement: S7 Fig — A bolus of iron tracer is administered in the plasma as radioactive NTBI (simulating an intravenous injection) or as radioactive duodenal iron (simulating intragastric gavage). Displayed are the fluxes to and from the NTBI species: duodenum to NTBI, NTBI to transferrin, and NTBI to liver. A- fluxes for intravenous administration in WT. B- fluxes for intravenous administration in HAMP-/-. C- fluxes for intragastric gavage in WT. D- fluxes for intragastric gavage in HAMP-/-. (TIF) [file pcbi.1006680.s011.tif]

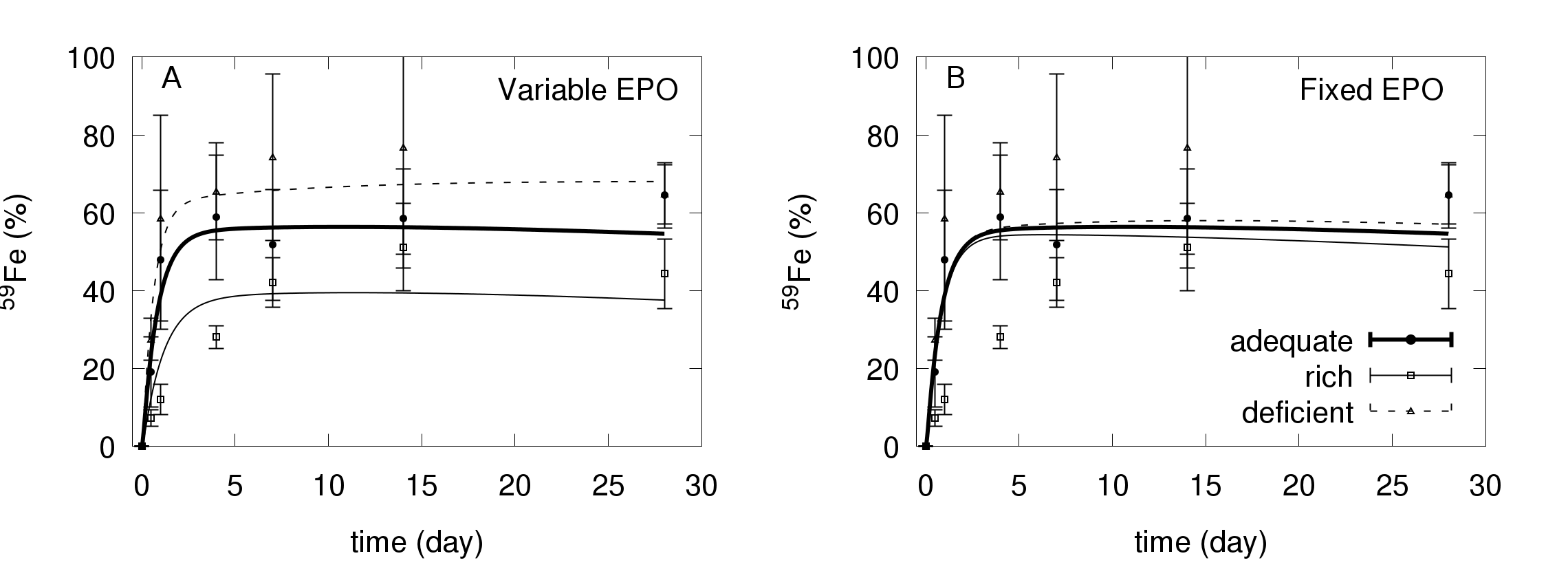

Supplement: S8 Fig — Panel A depicts the full model which includes the intact negative feedback loop between FeRBC and EPO (same as Fig 1B). Panel B depicts the behavior of the model when EPO is made unresponsive to FeRBC ([EPO] fixed), thus removing the negative feedaback loop. Without the loop the model is unable to incorporate enough 59Fe in RBC in the deficient diet, while in the rich diet the model incorporates too much 59Fe in RBC. (TIF) [file pcbi.1006680.s012.tif]
